# A Rare Case of *Cyberlindnera jadinii* Fungemia in a Kidney Transplant Recipient: A Case Report

**DOI:** 10.1155/crit/3478928

**Published:** 2025-07-28

**Authors:** Jayaditya Devpal Patil, Hiroshi Sogawa, David Mulligan

**Affiliations:** ^1^Department of Surgery, Yale New Haven Hospital, New Haven, Connecticut, USA; ^2^Division of Transplantation in the Department of Surgery, Yale New Haven Hospital, New Haven, Connecticut, USA

**Keywords:** *Cyberlindnera jadinii*, fungemia, immunosuppression, kidney transplant, prophylaxis

## Abstract

Despite revolutionary improvements in mortality, cardiovascular events, and quality of life, kidney transplantation has been plagued with perioperative mortality, graft rejection, and immunosuppression-associated complications, most commonly infections. Fungal infections in particular only account for 5% of all cases but have the worst outcomes, with mortality ranging between 25% and 80% in solid organ transplant recipients. *Candida*, *Cryptococcus*, and *Aspergillus* species are the commonest causative agents, and isolated cases of other species have been reported in other immunosuppressed pathologies. Here, we present the first recorded case of *Cyberlindnera jadinii* fungemia in a kidney transplant recipient, highlighting the need for close surveillance, early diagnosis, and potentially continuing posttransplant antifungal prophylaxis beyond the current recommended duration.

## 1. Introduction

Kidney transplantation (KT) has revolutionized the management of end-stage renal disease (ESRD), leading to lower mortality, reduced cardiovascular events, and improved quality of life compared with chronic dialysis [1]. Despite numerous benefits, KT is plagued with perioperative mortality, graft rejection, and immunosuppression-associated complications.

Infections are a major source of morbidity and mortality in kidney transplant recipients (KTRs) due to long-term immunosuppression. Fungal infections account for approximately 5%–10% of all infections in KTRs, with associated mortality ranging between 25% and 50% in cases of invasive infections [2]. Systemic mycosis is a significant problem in transplant patients and remains the major cause of death [3]. The incidence of fungal infections in solid organ transplant recipients (SOTRs) is the highest in the first 6 months posttransplant; however, delayed presentation may also occur [4]. Early infections are associated with surgical procedures, prolonged antibiotic use, and prolonged use of central catheters, while late infections are commonly secondary to environmental exposure or reactivation of latent infections [2].

Fungal infections in SOTRs are influenced by host and environmental factors. Host-related factors encompass age, gender, comorbidities, and immunosuppression, while environmental factors include exposure to sources of fungal contamination and suboptimal hygiene [5]. *Candida* and *Cryptococcus* species are the most frequently isolated yeasts, while filamentous fungi commonly include *Aspergillus* species [4]. Fungal infections also vary geographically, where *Histoplasma capsulatum* is commonly encountered in Ohio and Mississippi River valleys, while *Coccidioides immitis* is endemic to southwestern United States [6–8]. *Aspergillus* infections are seen globally [2, 6–8].

*Cyberlindnera jadinii* (*CJ*) is an example of a “non-*Saccharomyces*” yeast and is the teleomorphic parental species for *Candida utilis* [9]. It has been extensively studied as a source of single-cell protein, having the ability to produce vitamins, organic acids, and proteins [10]. However, no literature is available on *CJ* infection in SOTRs. Here, we present the first reported case of *CJ* infection in a KTR. The patient has consented to the use of data and case details for the purpose of research and publication.

## 2. Case Presentation

The patient is a 66-year-old female with a history of asthma, hypertension, insulin-dependent Type 2 diabetes mellitus, and ESRD, requiring a deceased donor kidney transplant in September 2023. She presented to the Yale New Haven Hospital (YNHH) emergency department (ED) in April 2024 with generalized fatigue, nausea, and vomiting with noncompliance to medications over 3 days. In the ED, potassium was 7 mEq/L (3.5–5 mEq/L), creatinine was 4.61 mg/dL (0.5–1.1 mg/dL), serum glucose was 340 mg/dL (70–140 mg/dL), and white cell count (WCC) was 5.7 × 10^9^/L (4.5–11 × 10^9^/L). An ultrasound of the renal graft showed elevated resistive indices with elevated peak systolic velocity at the arterial anastomosis but no hydronephrosis. However, a 1.1-cm perinephric fluid collection adjacent to the lower pole was also observed. Upon evaluation by the transplant surgery team, the patient became lethargic and acidotic, with tachypnea, requiring admission to the surgical intensive care unit (SICU) for monitoring. A full septic assessment was performed, and IV piperacillin–tazobactam 2.25 mg QID and creatinine dose-adjusted IV vancomycin were commenced along with a 5-day course of IV remdesivir 100 mg daily and a prednisone taper in the setting of a recent SARS COVID-19 infection prior to presentation. A respiratory viral panel was positive for human metapneumovirus. Additional viral panel workup was negative for Epstein–Barr virus (EBV) and BK virus; however, it was positive for *Cytomegalovirus* (CMV). On review, the patient had persistently positive CMV PCR with the most recent quantitation value of 41 IU/mL. The patient's home immunosuppression regimen of tacrolimus (PO 7 mg in the morning and 8 mg in the evening) and mycophenolate mofetil (MMF, PO 250 mg BID) and prophylaxis regimen of valganciclovir (PO 450 mg daily) was continued, and additional prophylaxis with atovaquone (PO 1.5 g daily) was commenced. Initial blood and urine cultures were both positive for *Klebsiella pneumoniae*. Prophylactic fluconazole (PO 100 mg daily) was commenced with repeat blood cultures growing extended-spectrum beta-lactamase (ESBL) *Klebsiella pneumoniae*. The patient remained afebrile with downtrending WCC throughout this course. The following day, one of two blood culture bottles was positive for *CJ*. At the time, the patient was started on IV ertapenem 100 mg daily, per infectious disease (ID) recommendation. Immunosuppression with MMF was held in the setting of the current infection, and further investigation for the infectious source with a transthoracic echocardiogram (TTE) was negative. Repeat blood cultures 48 h later continued to grow *CJ* from one of the two bottles, and IV anidulafungin 100 mg daily was commenced. Further assessment with cryptococcal serum antigen and stool microsporidium were both negative. The first negative blood culture was identified 1 week after the initial presentation.

Concern for invasive fungal collection prompted a positron emission tomography-computer tomography (PET CT) scan showing radiotracer-avid tubular elongated structure crossing the inferior aspect of the transplanted kidney. Due to poor anidulafungin penetration of the genitourinary system, a single dose of IV weight-based amphotericin B (0.3 mg/kg) was administered with the goal of reducing fungal burden and offsetting significant kidney injury. Two weeks after the initial presentation, the minimum inhibitory concentration (MIC) reports were released: voriconazole 2, amphotericin B 0.25, fluconazole 64, and anidulafungin < 0.015. The ID team recommended ertapenem continual for an additional week and anidulafungin for three additional weeks. With clinical improvement, the patient was discharged to a short-term rehab (STR) facility.

The patient represented to the ED a week after discharge in May 2024, with diabetic ketoacidosis and an elevated WCC to 21 × 10^9^/L (4.5–11 × 10^9^/L). The patient was commenced on intravenous insulin with a complete septic assessment. The patient was restarted on IV ertapenem 100 mg daily and received one dose of creatinine-adjusted IV vancomycin. Blood cultures from admission were positive for ESBL *Klebsiella pneumoniae*. A repeat TTE was negative. Magnetic resonance imaging of the spine was performed to assess for an infectious source but was negative. Blood culture showed no growth; however, a chest x-ray was suggestive of aspiration pneumonia, and a 5-day course of PO ciprofloxacin 500 mg BID was started. The patient completed her course of ertapenem and was discharged to STR. She was closely followed by ID and continued valganciclovir until at least two nonquantifiable CMV PCR results. The patient has since been well and was recently seen in the postrenal transplant clinic in March 2025, where no symptoms referable to renal transplant were reported, with a plan for follow-up in 3 months.

## 3. Discussion

This extremely rare case of *CJ* fungemia in a KTR follows a complex course of bacteremia, aspiration pneumonia, fungemia, and diabetic ketoacidosis requiring readmission. This case identifies a patient with fungemia 7 months post KT, an uncommon period during which fungal infections account for the lowest rates of invasive infections and antifungal agents are not routinely recommended beyond 6 months post KT [4]. However, given the high mortality and poor outcomes in fungal infections, prompt investigation and treatment are mandated.

Demographics of fungal infections vary, and although no epidemiological study was conducted specifically assessing *CJ* distribution in the New England region, one study reviewing candidemia in the state of Connecticut across two study periods did report significant improvements in patient survival [11]. Although this does not provide information on the infectious source, our patient did not report any recent travel outside of the state prior to admission.

Antifungal prophylaxis regimens vary across institutes. One meta-analysis of 14 randomized controlled trials evaluated the overall efficacy of antifungal prophylaxis in SOTRs and concluded that antifungal prophylaxis did not reduce mortality [12]. In KTRs, neither ketoconazole nor cotrimoxazole significantly reduced invasive fungal infections. Gaps in antifungal coverage should be noted for echinocandins and azoles, as they lack activity against *Cryptococcus neoformans* and molds, respectively [13]. At our institute, fluconazole is frequently used as prophylaxis owing to its safety profile and limited nephrotoxicity [12].

Isolated cases of *CJ* have been reported in nontransplant patients. One case involves *CJ* fungemia in an aplastic anemia patient treated with caspofungin and amphotericin B, eventually requiring bone marrow transplantation with full recovery [14]. The immunosuppressed state of transplant patients presents a unique scenario with inadequate inflammatory response, owing to a poor prognosis.

## 4. Conclusion

This rare case of invasive *CJ* infections highlights the importance of prompt diagnosis of fungemia and the infective potential beyond the 6-month posttransplantation window. In such rare cases, adapted supportive care and antifungal treatment are essential. It is also worthwhile to reconsider antifungal prophylaxis in the first 6 months post transplantation, ensuring to provide adequate coverage.

## Data Availability

Data sharing is not applicable to this article as no new data were created or analyzed in this study.
